# Hypopigmented Patches in an Adolescent: A Diagnostic Dilemma and the Value of Clinical Restraint

**DOI:** 10.7759/cureus.107596

**Published:** 2026-04-23

**Authors:** Sri Naidnur, Coral Martes-Villalobos, Kevin Burningham, Bryan Gammon, Soham Chaudhari, Rick Lin

**Affiliations:** 1 Dermatology, Oasis Dermatology Group, McAllen, USA; 2 Dermatology, Universidad Central del Caribe School of Medicine, Bayamon, PRI; 3 Dermatology, Hospital Corporation of America (HCA) Healthcare Corpus Christi Medical Center Bay Area, McAllen, USA; 4 Dermatopathology, Sagis Diagnostics, Houston, USA; 5 Dermatology, Marshall University Joan C. Edwards School of Medicine, Huntington, USA

**Keywords:** clinical restraint, clinicopathologic correlation, diagnostic dilemma, hypopigmented lesions, pediatric dermatology, post-inflammatory hypopigmentation, progressive macular hypomelanosis, psychosocial distress, vitiligo mimic, wood’s lamp

## Abstract

Hypopigmented lesions in pediatric patients can present a diagnostic challenge, particularly when clinical and histopathologic findings do not clearly support a single diagnosis, and this may be further complicated in individuals with skin of color, where distinguishing hypopigmented from depigmented lesions can be more difficult.

A 12-year-old Hispanic female presented with long-standing, progressive hypopigmented patches involving the face, neck, and torso. Vitiligo was initially suspected based on clinical appearance; however, Wood’s lamp and histologic findings argued against a definitive diagnosis. The biopsies favored post-inflammatory hypopigmentation, although the clinical history was not entirely supportive. Progressive macular hypomelanosis was considered a diagnosis of exclusion based on the overall clinical context. The patient and family pursued counseling during the course of evaluation, after which she developed greater self-confidence, embraced her skin findings, and declined further treatment.

This case highlights the importance of clinicopathologic correlation and diagnostic restraint when evaluating benign hypopigmented lesions in pediatric patients. Premature labeling of non-vitiliginous conditions as vitiligo may have significant psychosocial implications, particularly in younger patients, and may contribute to increased distress for both patients and their families.

## Introduction

Disorders of pigment loss can be broadly divided into hypopigmented and depigmented conditions [1]. Vitiligo is an acquired autoimmune depigmenting disorder characterized by loss of melanocytes, whereas post-inflammatory hypopigmentation, pityriasis alba, and progressive macular hypomelanosis (PMH) involve reduced melanin with preservation of melanocytes [1-5]. Clinically, depigmented lesions such as vitiligo tend to present as well-demarcated, chalk-white patches, whereas hypopigmented conditions often appear as lighter, less sharply defined areas with more subtle contrast [1]. In pediatric patients, these conditions may appear similar, making diagnosis challenging, particularly in individuals with skin of color, where contrast differences, difficulty distinguishing hypopigmented from depigmented lesions, and the higher prevalence of overlapping pigmentary conditions can complicate clinical evaluation [1].

Additionally, congenital or early-onset hypopigmented lesions such as nevus depigmentosus and nevus anemicus may be considered in pediatric patients; however, they are usually stable over time and can be distinguished clinically, as nevus depigmentosus presents as a persistent lighter patch with decreased pigment, while nevus anemicus appears lighter than the surrounding skin and blends in with pressure due to underlying vascular constriction [1,3].

PMH is a benign pigmentary disorder most often seen in adolescents and young adults and has been reported more frequently in females and in patients with skin of color [1,5]. It presents with ill-defined, non-scaly hypopigmented macules, typically on the trunk, and often shows minimal or nonspecific histopathologic findings with preserved melanocytes [1,5]. In this case, the clinical and histologic findings did not clearly fit a single diagnosis, and PMH was considered a working diagnosis, highlighting the importance of clinicopathologic correlation and clinical restraint.

## Case presentation

A 12-year-old Hispanic female with Fitzpatrick skin type III presented with long-standing hypopigmented patches involving the face, neck, and upper torso. The lesions were first noted on the face during infancy and gradually increased in size and distribution over time. They were asymptomatic. There was no clear history of preceding inflammation, trauma, or infection, although subclinical inflammation could not be excluded. The patient had no history of atopy, vitiligo, or autoimmune disease in the family, and no significant sunburns or prior topical treatments. A sibling was noted to have similar hypopigmented skin findings.

**Figure 1:**
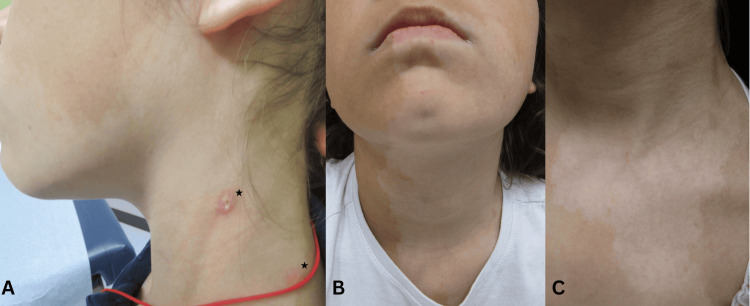
Clinical presentation of ill-defined hypopigmented patches. (A) Left lateral profile demonstrating involvement of the lateral neck, extending superiorly beyond the mandibular border; prior punch biopsy sites are visible (black stars). (B) Anterior view demonstrating involvement extending beyond the submental region into the anterior neck. (C) Close-up of the anterior neck and upper chest. Written, signed consent from the parents, permitting disclosure of the patient’s identity in an open-access publication, was provided to the journal.

**Figure 2:**
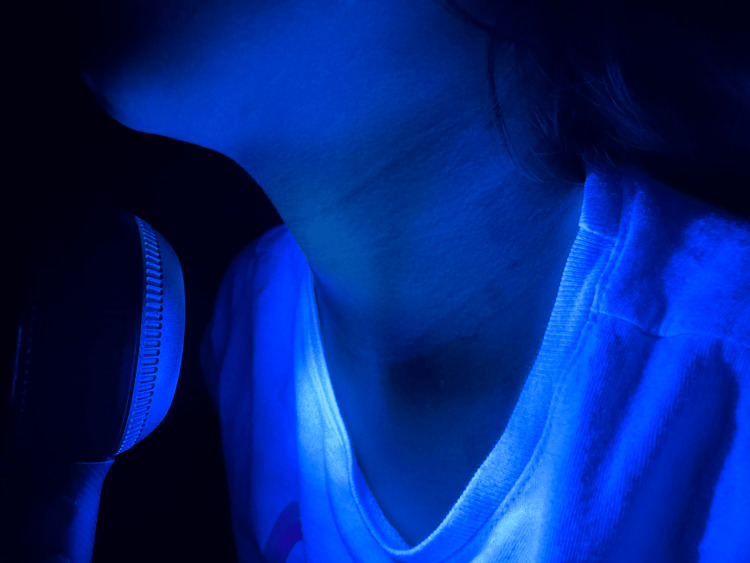
Wood’s lamp examination of the anterior neck. Absence of bright, chalky-white accentuation and red follicular fluorescence.

On examination, ill-defined hypopigmented patches were noted on the face, neck, and trunk, without scale or surface changes (Figure 1A-1C). Initial clinical differential diagnoses included vitiligo, tinea versicolor, post-inflammatory hypopigmentation, pityriasis alba, seborrheic dermatitis, and, less likely, hypopigmented mycosis fungoides. Vitiligo was initially suspected. Wood’s lamp examination did not demonstrate bright, chalky-white accentuation or red follicular fluorescence (Figure 2). Dermoscopy was not performed at the time of evaluation.

**Figure 3:**
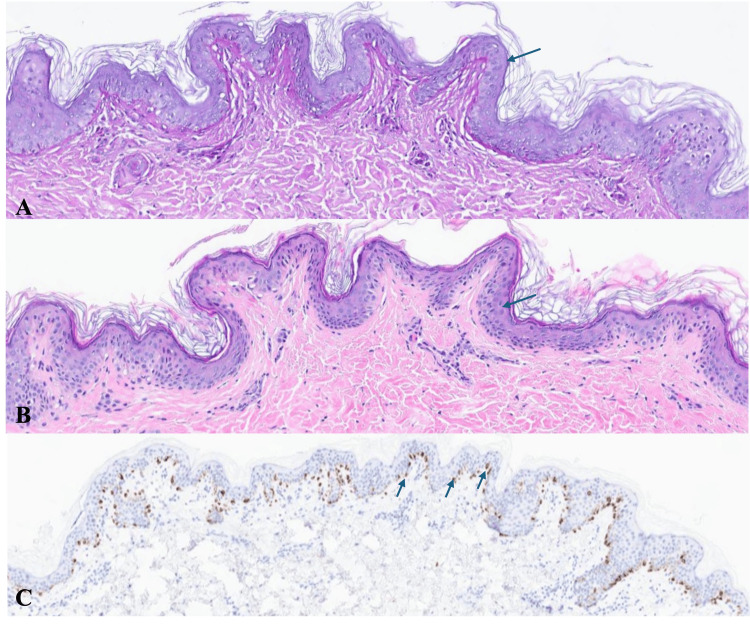
Histopathologic images of the second punch biopsy from the left lateral neck. (A) Periodic acid–Schiff stain demonstrating no fungal organisms within the stratum corneum (blue arrow). (B) Hematoxylin and eosin ×4 stain showing preserved epidermal architecture with mild spongiosis within the epidermis (blue arrow). (C) MART-1 immunohistochemical stain highlighting a preserved distribution of melanocytes along the basal layer of the epidermis (blue arrows), consistent with a non-depigmenting process.

Two 4-mm punch biopsies were obtained one month apart from different locations on the left neck. Clinical and histopathologic details are summarized in Table 1, demonstrating preserved melanocytes with nonspecific pigmentary alteration. Histology (Figure 3A-3C) favored post-inflammatory hypopigmentation, with pityriasis alba also considered on the second biopsy.

**Table 1:** Summary of punch biopsy findings.

Feature	1st punch biopsy (left posterior neck, 4 mm)	2nd punch biopsy (left lateral neck, 4 mm)
PAS stain	Negative for fungi	Negative for fungi
MART-1	Preserved basal melanocytes	Preserved basal melanocytes
Melanin pigment	Decreased	Decreased
Melanophages	Present in the superficial dermis	Present
Spongiosis	Not identified	Mild focal spongiosis
Interpretation	Post-inflammatory hypopigmentation favored	Post-inflammatory hypopigmentation; pityriasis alba considered with clinical correlation

Given clinicopathologic discordance, the dermatology provider consulted dermatopathology and shared clinical images for further correlation. One month following the second biopsy, additional Fontana-Masson staining demonstrated patchy decreased melanin within basal keratinocytes, favoring PMH as a diagnosis of exclusion.

Management evolved alongside diagnostic uncertainty, including trials of topical calcineurin inhibitors, benzoyl peroxide with clindamycin, and a brief course of phototherapy; however, after counseling, the patient accepted her skin findings and declined further treatment.

Given the overall clinical and histopathologic findings, a single definitive diagnosis could not be established. PMH was considered a working diagnosis of exclusion, and observation without active treatment was elected.

## Discussion

Vitiligo was initially considered in this case; however, the absence of bright, chalky white accentuation on Wood’s lamp examination and the preservation of melanocytes on repeated biopsies argued against a definitive diagnosis [1,2].

Histopathologic findings from both biopsies demonstrated preserved melanocytes with nonspecific pigmentary alteration, favoring post-inflammatory hypopigmentation. Mild spongiosis in one specimen raised the possibility of pityriasis alba, although this finding is subtle and must be interpreted in the clinical context [1,3,4].

The overall clinical picture was less consistent with pityriasis alba or post-inflammatory hypopigmentation. Pityriasis alba typically presents with facial involvement, subtle scaling, and a fluctuating course. In contrast, this patient demonstrated a long-standing pattern with gradual extension beyond the face to the neck and trunk without appreciable scale [1,3,4]. Post-inflammatory hypopigmentation usually follows a preceding inflammatory event, which was not clearly identified in this case, although subclinical inflammation cannot be excluded [1,3,4].

Congenital or early-onset hypopigmented lesions, such as nevus depigmentosus and nevus anemicus, may also be considered retrospectively given their early onset and distribution; however, their typically stable nature makes them less consistent with this presentation [1].

PMH was considered a working diagnosis of exclusion. Although PMH lacks strict, universally accepted diagnostic criteria, it was favored in this case given the clinical course, distribution, absence of scale, and histopathologic findings demonstrating decreased melanin with preserved melanocytes [1,5]. While facial involvement is not classic, the overall clinicopathologic correlation favored PMH over other entities in the differential diagnosis. Taken together, the clinical progression, distribution, absence of scale, and histopathologic findings of decreased melanin with preserved melanocytes most strongly support PMH as the working diagnosis.

The pathophysiology of PMH is not fully understood. Still, it has been associated with Cutibacterium acnes, which may contribute to altered melanogenesis by producing porphyrins and other factors that affect melanocyte function [5]. This association may explain the red follicular fluorescence observed in some cases under Wood’s lamp examination. However, this finding was not present in our patient and does not exclude the diagnosis [5]. Treatment approaches are not standardized but often include topical antimicrobials such as benzoyl peroxide or clindamycin, sometimes in combination with phototherapy, with variable response [1,5,6].

Key distinguishing clinical and histopathologic features among the primary diagnostic considerations are summarized in Table 2. Several conditions in this differential lack definitive diagnostic criteria, emphasizing the importance of clinicopathologic correlation.

**Table 2:** Comparison of key differential diagnoses. PAS, Periodic acid-Schiff Table Credit: Sri Naidnur. Data compiled and synthesized from previously published sources [1-5].

Condition	Epidemiology	Clinical features	Wood’s lamp	Histopathology
Vitiligo	Any age; autoimmune; ± family history	Well-demarcated depigmented patches	Bright chalky-white accentuation	Loss of melanocytes
Post-inflammatory hypopigmentation	Any age; follows inflammation	Ill-defined hypopigmented patches at prior sites	Variable	Decreased melanin with dermal melanophages; melanocytes typically preserved
Pityriasis alba	Children/adolescents; often atopic	Ill-defined hypopigmented patches, often facial; subtle scale	Accentuation without fluorescence	Mild spongiosis; preserved melanocytes
Tinea versicolor	Adolescents/young adults	Hypo-/hyperpigmented patches with fine scale, trunk/neck	Variable yellow-green/gold fluorescence	Hyphae and spores in stratum corneum (PAS+)
Progressive macular hypomelanosis	Adolescents; more common in females and skin of color	Ill-defined, non-scaly hypopigmented patches on trunk ± extension	Red follicular fluorescence may be present	Decreased melanin; preserved melanocytes

Hypopigmented mycosis fungoides, a variant of cutaneous T-cell lymphoma, was considered less likely given the absence of epidermotropism, atypical lymphocytes, and a stable clinical course [1]. Additionally, early infancy onset is not characteristic of this entity, further supporting a benign etiology [1].

This case highlights a working diagnosis of PMH while emphasizing real-world, patient-centered management. A multidisciplinary approach, including mental health support when needed, can help patients accept their skin findings, as medical treatments are not always necessary to achieve a satisfactory outcome.

## Conclusions

This case highlights the diagnostic complexity of pediatric hypopigmented lesions, where clinical and histopathologic findings may overlap and lack definitive criteria. In our patient, preserved melanocytes, nonspecific biopsy findings, and an atypical clinical distribution made a single unifying diagnosis challenging, with PMH favored as a diagnosis of exclusion.

This case underscores the importance of clinicopathologic correlation and diagnostic restraint when evaluating benign pigmentary disorders. Premature labeling of such conditions as vitiligo may carry unnecessary psychosocial implications, particularly in younger patients and in individuals with skin of color.

Equally important, this case emphasizes the role of patient preference and a multidisciplinary approach to care. Following psychological counseling, the patient was encouraged to view her condition positively and subsequently developed greater self-confidence, accepted her skin's appearance, and declined further medical management, highlighting that supportive care and reassurance may be as valuable as medical treatment in selected cases.
